# Regulation of endothelial permeability and transendothelial migration of cancer cells by tropomyosin-1 phosphorylation

**DOI:** 10.1186/2045-824X-4-18

**Published:** 2012-11-17

**Authors:** Bryan Simoneau, François Houle, Jacques Huot

**Affiliations:** 1 Centre de recherche du CHU de Québec, l’Hôtel-Dieu de Québec et Le Centre de recherche en cancérologie de l’Université Laval, 9 rue McMahon, Québec, G1R 2J6, Canada

**Keywords:** Tropomyosin phosphorylation, Permeability, Oxidative stress, Transendothelial migration

## Abstract

**Background:**

Loss of endothelial cell integrity and selective permeability barrier is an early event in the sequence of oxidant-mediated injury and may result in atherosclerosis, hypertension and facilitation of transendothelial migration of cancer cells during metastasis. We already reported that endothelial cell integrity is tightly regulated by the balanced co-activation of p38 and ERK pathways. In particular, we showed that phosphorylation of tropomyosin-1 (tropomyosin alpha-1 chain = Tm1) at Ser283 by DAP kinase, downstream of the ERK pathway might be a key event required to maintain the integrity and normal functions of the endothelium in response to oxidative stress.

**Methods:**

Endothelial permeability was assayed by monitoring the passage of Dextran-FITC through a tight monolayer of HUVECs grown to confluence in Boyden chambers. Actin and Tm1 dynamics and distribution were evaluated by immunofluorescence. We modulated the expression of Tm1 by siRNA and lentiviral-mediated expression of wild type and mutated forms of Tm1 insensitive to the siRNA. Transendothelial migration of HT-29 colon cancer cells was monitored in Boyden chambers similarly as for permeability.

**Results:**

We provide evidence indicating that Tm1 phosphorylation at Ser283 is essential to regulate endothelial permeability under oxidative stress by modulating actin dynamics. Moreover, the transendothelial migration of colon cancer cells is also regulated by the phosphorylation of Tm1 at Ser283.

**Conclusion:**

Our finding strongly support the role for the phosphorylation of endothelial Tm1 at Ser283 to prevent endothelial barrier dysfunction associated with oxidative stress injury.

## Introduction

By gating the traffic of molecules and cells across the vessel wall, vascular endothelial cells play an active role in regulating cardiovascular and systemic homeostasis and in modulating physiopathological processes such as inflammation and immunity [1-3]. The functions of endothelial cells are regulated by physiological agonists and stress stimuli in a process called *endothelial cell activation*. This process is associated with the induction of intracellular signalling pathways that converge on the stimulation of constitutive functions of the endothelium, the synthesis and release of mediators of inflammation, the induction of adhesion molecules, and with structural changes in the actin cytoskeleton that govern motility and permeability [4-6]. Dysfunction of the regulatory systems of the endothelium and its incapacity to cope with its physicochemical surrounding lead to disruption of endothelial integrity and genesis of cardiovascular pathologies including atherosclerosis, hypertension and coagulation abnormalities [7,8].

Endothelial cells are heavily exposed to Reactive Oxygen Species (ROS) and these latter are major regulators of physiological and pathological processes involving the endothelium. Notably, endothelial cells produce ROS in response to ischemia-reoxygenation. In turn, ROS contribute to the modulation of several signalling pathways including calcium-dependent signalling as well as pathways regulating vascular permeability [9-12]. Importantly, loss of endothelial cell integrity and selective permeability barrier is an early event in the sequence of oxidant-mediated injury and may result in atherosclerosis, hypertension and facilitation of transendothelial migration of cancer cells (TEM) during metastasis [13-16]. It is thus of high clinical importance to better understand how ROS regulate endothelial permeability in normal and pathological situations. Along these lines, the current paradigm is that endothelial cell permeability is regulated by the opposing centripetal-centrifugal forces associated with cytoskeletal dynamics [17]. The equilibrium between the centripetal and centrifugal forces is under the regulation of different signalling pathways. Notably, we reported that endothelial cell integrity is tightly regulated by the balanced co-activation of p38 and ERK pathways [18]. In particular, we showed that in response to E-selectin activation, endothelial cell permeability and TEM of cancer cells are regulated by the formation of stress fibers by p38-dependent actin polymerization and myosin light chain phosphorylation and by ERK-dependent regulation of the VE-cadherin-βcatenin complex at the adherens junctions [5]. In oxidative stress conditions, the strong actin-polymerizing activity generated through the p38/MAKAP kinase-2/HSP27 axis is associated with membrane blebbing when the ERK pathway is inhibited [19,20]. The blebbing activity caused gaps in the endothelium layers, which were due to an impairment of the phosphorylation of tropomyosin-1 (RecName: Full= Tropomyosin alpha-1 chain, herein named Tm1) at Ser283 by DAP kinase, downstream of the ERK pathway [19,21]. This suggests that phosphorylation of tropomyosins might be a key event required to regulate their functions. Along these lines, phosphorylation of Tm1 at Ser283 is required for the formation of stress fibers and thereby contraction of the endothelial cells [21]. On the other hand, phosphorylation of TM2 at S61 occurs downstream of the PI3K pathway and is required for the internalization of the beta-adrenergic receptor [22]. Moreover, Tm1 kappa, a splice variant of Tm1α gene, is increased in patients with chronic dilatated cardiomyopathy and it modulates the sensitivity of the myofilaments in a phosphorylation-dependent manner [23]. The *in vivo* functions of Tms are not necessarily direct and may require other actin binding proteins such as caldesmon and HSP27 [24].

In the present study, we investigated the role of Tm1 in regulating endothelial cell permeability and TEM of colon cancer cells in response to oxidative stress. We show for the first time that phosphorylation of Tm1 at Ser283 is a major regulator of both endothelial cell permeability and TEM in response to oxidative stress. In particular, we provide evidence indicating that phosphorylated Tm1 contributes to maintain the integrity of the endothelium under oxidative stress condition by its crucial participation to the remodelling of actin cytoskeleton into stress fibers. These finding strongly support the role for the phosphorylation of Tm1 at Ser283 to prevent oxidative stress injury associated with endothelial barrier dysfunction.

## Materials and methods

### Chemicals

H_2_O_2_ and endothelial cell growth supplement (ECGS) were from Sigma-Aldrich (Oakville, On, Canada). PD098059 was purchased from Calbiochem (San Diego, CA) and was diluted in DMSO to make stock solutions of 20 mM. Histamine and NaF were purchased from Sigma-Aldrich. Histamine was diluted in water to make stock solutions of 5 mM. Chemicals for electrophoresis were obtained from Bio-Rad (Mississauga, On, Canada) and Fisher Scientific (Montréal, Qc, Canada).

### Cells

HUVECs were isolated by collagenase digestion of umbilical veins from undamaged sections of fresh cords [20]. The cords were obtained after approbation by the CRCHUQ ethical committee. Subcultures were maintained in EGM2 media (LONZA, Allendale, NJ, USA). Replicated cultures were obtained by trypsinization and were used at passages <5. Human Embryonic Kidney cells (HEK293T) were cultivated in DMEM containing 10% foetal bovine serum (FBS) and antibiotics. Human colorectal adenocarcinoma cells (HT29) were cultivated in McCoy’s 5A medium supplemented with 10% FBS. Cultures were maintained at 37°C in a humidified atmosphere containing 5% CO_2_.

### Antibodies

Anti-tropomyosin (clone TM311) monoclonal and anti-α-tubulin mouse monoclonal (clone B-512) antibodies were purchased from Sigma. Anti-living color (GFP) rabbit polyclonal antibody was purchased from BD Biosciences (Mississauga, On, Canada). Anti-mouse-IgG-horseradish peroxidase (HRP) and anti-rabbit-IgG-HRP antibodies were from Jackson Laboratory (Bar Harbor, ME, USA). Anti-ERK2 is a rabbit polyclonal antibody raised against a synthetic peptide that corresponds to the 14 C-terminal amino acids of rat ERK2 [20].

### Plasmids and small interfering RNA (siRNA)

Tropomyosin-1 cDNA (RecName :Full= Tropomyosin alpha-1 chain, herein named Tm1) was cloned by PCR amplification from IMAGE clone 562592 (ATCC) into pIRES-hrGFP2a [21]. The tropomyosin-1 S283A was generated by PCR site-directed mutagenesis on pIRES-hrGFP2a-tropomyosin-1 construct using the primers 5^′^-ATGACTGCTATATAACTCGAGTACCCATATGACG-3^′^ and 5^′^-TTATATAGCAGTCATATCGTTGAGAGCGTGG-3^′^[21]. Validated Tm1 siRNA #7 (Hs_TPM1_7) was purchased from QIAGEN (Mississauga, On, Canada) and was designed to target the mRNA of human Tm1 (GenBankTM accession no. NM_ 000366.1). The target sequence of Tm1 siRNA #7 is as follows: sense, 5^′^-GAGUGAGAGAGGCAUGAAATT-3^′^ and antisense, 5^′^-UUUCAUGCCUCUCUCACUCTC-3^′^. Non-targeting silencer negative control siRNA #1 was purchased from Ambion (Austin, TX, USA). The tropomyosin-1 wild-type (Tm1wt) and tropomyosin-1 S283A (Tm1S283A) constructs were rendered insensitive to Tm1 siRNA #7 by PCR-mediated mutagenesis at the sites indicated in bold in the mutagenic oligonucleotides: 5^′^-GAG**C**GAG**C**GAGG**G**ATGAA**G**GTCATTGAG-3^′^ (sense) and 3^′^-GTCTACTCTC**G**CTC**G**CTCC**C**TACTT**C**C-5^′^ (reverse). Plasmids necessary for lentiviral particles production were a kind gift of Dr. Manuel Caruso (Laval University, Qc, Canada). CSII-EF-MCS-IRES2-Venus (CSII-Venus) vector was a kind gift of Dr. Hiroyuki Miyoshi (RIKEN Tsukuba Institute, Japan). Tm1S283A insensitive to siRNA #7 was subcloned in CSII-Venus vector using NotI and BamHI restriction sites (CSII-Venus-Tm1S283A). The CSII-Venus-Tm1wt construct insensitive to siRNA #7 was generated by PCR site-directed mutagenesis on CSII-Venus-Tm1S283A construct using the following primers: sense, 5^′^-TATGACTTCCATATAAGGATCCGCCCCTCTCCCTCCCCC-3^′^ and antisense, 5^′^-GCGGATCCTTATATGGAAGTCATATCGTTGAGAGCGTGGTCCAG-3^′^. KOD Hot Start DNA polymerase was used according to the manufacturer protocol (EMD, Philadelphia, PA, USA).

### Lentiviral particle preparation

HEK293T were plated at 3×10^6^ per 100 mm Petri dish in 10 ml DMEM supplemented with 10% FBS. Co-transfection was performed by Ca^2+^ phosphate-mediated transfection using 2.5 μg of pRSV-Rev, 6.5 μg of pMDLg-pRRE, 3.5 μg of pMD2.G together with 10 μg of transgene expressing vectors (CSII-Venus, CSII-Venus-Tm1wt or CSII-Venus-Tm1S283A). After overnight incubation, media were replaced with 5 ml lentiviral particle collection media (M199 medium containing 20% heat-inactivated FBS, ECGS, L-glutamine, heparin and antibiotics). After 24 hours, the media were collected and centrifuged at 1200 g, for 5 minutes at 4°C. The supernatant was stored in aliquots at −80°C. The Multiplicity of Infection (MOI), known as the ratio of infectious virus particles per cell, was determined after a 48 hours transduction of HUVECs in cascade dilution experiment. The percentage of GFP positive cells was determined by FACS using EPICS-XL-MCL flow cytometer (Beckman-Coulter, Ramsey, MN, USA) [25,26].

### Transfection and transduction

HUVECs and HEK293T were transfected with siRNA or plasmid vectors using X-tremeGene HP Transfection Reagent obtained from Roche (Laval, Qc, Canada) according to the manufacturer’s protocol or by electroporation [21]. HUVECs were transduced or not with CSII-Venus (empty vector) or CSII-Venus-Tm1wt (Tm1 wild-type vector) or CSII-Venus-Tm1S283A (non-phosphorylatable Tm1 mutant vector) using lentivirus-mediated infection in the presence of 8 μg/ml hexadimethrine bromide (Sigma-Aldrich, Oakville, On, Canada).

### Western blotting

After treatment, cells were lysed using SDS-PAGE loading buffer. Equal amounts per well of total proteins were separated by SDS-PAGE, and the gels were transferred onto nitrocellulose membranes for western blotting. After incubating nitrocellulose membranes with the appropriate primary antibodies, antigen-antibody complexes were detected with an anti-IgG antibody coupled to horseradish peroxidase and then revealed using an enhanced chemiluminescence kit. Quantification of immunoreactive bands was done by densitometric scanning using the Image J software.

### Endothelial permeability assay

Permeability across endothelial cell monolayers was measured using gelatin-coated Transwell units (Boyden chambers) [6.5 mm diameter, 0.4 μm pore size polycarbonate filter; Corning Costar; Pittston, PA, USA]. Transfection and transduction--First day, HUVECs were plated at a density of 3×10^5^ cells per 60 mm Petri dishes and were cultured for 24 hours. Second day, exponentially growing HUVECs were transduced or not with CSII-Venus (empty vector) or CSII-Venus-Tm1wt (Tm1 wild-type vector) or CSII-Venus-Tm1S283A (non-phosphorylatable Tm1 mutant vector) using lentivirus-mediated infection in the presence of 8 μg/ml hexadimethrine bromide. After 24 hours, infected or non-infected HUVECs were transfected or not (controls) with Tm1 siRNA #7 using X-tremeGene HP Transfection Reagent according to the manufacturer’s protocol. The next day, HUVECs were plated at a density of 0.6×10^5^ cells (or 1×10^5^ in the case of transduced cells) per well (upper chamber) and were cultured for 3 days until the formation of a tight monolayer. Endothelial permeability was determined by measuring the passage of FITC-labelled dextran (1 mg/ml of fluorescein isothiocyanate-dextran, molecular mass: 40 kDa; Sigma-Aldrich) through the HUVEC monolayer. Thereafter, NaF (1 mM for 1 hour) was added to the incubation medium (upper chamber) to inhibit phosphatases and allow the accumulation of phosphorylated tropomyosin-1. Then, H_2_O_2_ was added to the upper chamber in the presence of 1 mg/ml FITC-labelled dextran. After 30 minutes of treatment, 100 μl was collected from the lower compartment and fluorescence was evaluated using a Fluoroskan Ascent Microplate Fluorometer following manufacturer’s protocol (Thermo Scientific, Mtl, Qc, Canada).

### Transendothelial cell migration assay

Cancer cell migration through endothelial barrier in response to H_2_O_2_ was studied in Boyden chambers (6.5 mm diameter, 5.0 μm pore size polycarbonate filter; Corning Costar; Pittston, PA, USA). HUVECs transduced and transfected as mentioned above (see section Endothelial permeability assay) were plated at a density of 1.0×10^5^ per well and were cultivated for 3 days until the formation of a tight monolayer. Thereafter, H_2_O_2_ was added to the upper chamber for 30 minutes. Then, EGM2 medium was removed and replaced by migration buffer (199 medium, 10 mM HEPES pH 7.4, 1 mM MgCl_2_, and 0.5% bovine serum albumin). HT-29 were harvested with trypsin, counted, centrifuged, and resuspended at 1×10^6^ cells/ml in McCoy’s medium (McCoy’s 5A medium supplemented with 10% FBS). Then, HT-29 were stained with calcein (500nM) for 30 minutes at 37°C, were centrifuged, and were resuspended at 1.5×10^6^ cells/ml in migration buffer. Cancer cells were added on the HUVEC monolayer at a density of 1.5×10^5^ cells per well. After 4 hours and a half of migration, cells in the upper chamber were removed with a cotton swab. Then, HT-29 cells that crossed the membrane of the Boyden chamber were counted in five different fields using a Nikon TE300 fluorescence microscope equipped with a 20x objective.

### Cell viability assay

Cell viability has been determined by using the Quick Cell Proliferation Assay Kit from BioVision. This cell proliferation and viability assay is based on the enzymatic cleavage of the tetrazolium salt WST-1 to formazan dye by cellular mitochondrial dehydrogenases that are active in viable cells only. The formazan dye produced by viable cells is quantified by measuring the absorbance of the dye solution at 440 nm. The exact procedure that was followed was as described in the BioVision protocol sheet.

### Fluorescence microscopy

Confocal microscopy was used for immunofluorescence visualization of F-actin, tropomyosin-1 and EGFP. HUVECs were plated on gelatin-coated microscope cover slides into 35 mm Petri dishes. After treatment, cells were fixed with 3.7% formaldehyde and permeabilized with 0.1% saponin in phosphate buffer, pH 7.5. F-actin was detected using Alexa-488-phalloidin (Invitrogen, Carlsbad, CA) diluted 1:400 in phosphate buffer. Tropomyosin-1 was detected using an anti-tropomyosin (clone TM311) mouse monoclonal antibody. Living color rabbit antibody was used for EGFP staining. The antigen-antibody complexes were detected with Alexa-568-mouse-IgG or Alexa-568-rabbit-IgG secondary antibodies. The cells were examined using Nikon Eclipse 600 microscope equipped with 20x and 40x objectives.

### Statistical analysis

Results are expressed as mean values ± SEM. Unless specified, the figures present results that were obtained from a representative experiment of at least three distinct experiments. The n value means the total number of replicates in this experiment. Unpaired Student *t* test was used for comparison between two means. A p-value <0.05 was considered as statistically significant.

## Results

### H_2_O_2_ induces the formation of transcytoplasmic stress fibers and increases endothelial cell permeability

In response to oxidative stress, activation of the p38 pathway favours actin polymerization, which contributes to cytoskeletal remodelling into stress fibers, which in turn, are required to regulate endothelial permeability [17,20]. Accordingly, inhibition of ROS-induced p38 activation dampens the increase in endothelial cell permeability [27]. Intriguingly, in the presence of ERK inhibition, the strong actin polymerization generated via the p38 pathway induced by ROS is associated with membrane blebbing, an early toxic manifestation of oxidative stress [18,28]. We provided evidence indicating that ROS-induced membrane blebbing resulted in an inhibition of ERK-dependent phosphorylation of Tm1 downstream of Death Associated-Protein Kinase-1 (DAPK1) [18,20,21,28]. This suggested that activation of the ERK-Tm1 axis might be a key determinant in maintaining the integrity of the endothelium in response to oxidative stress. In this context, we investigated herein the role of the ERK-Tm1 axis in regulating endothelial cell permeability in response to ROS.

**Figure 1:**
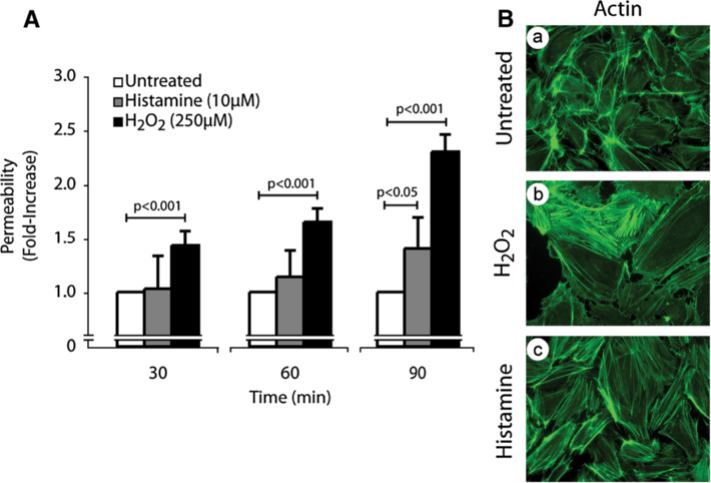
**Oxidative stress induces actin remodelling, disruption of endothelial layer integrity and increase of endothelial permeability.A**) HUVECs were plated in the upper part of a Boyden chamber at a density of 0.6×10^5^ per well and were cultivated for 3 days until the formation of a tight monolayer. Then, FITC-labelled dextran was added (1 mg/ml) together or not with H_2_O_2_ (250 μM) or histamine (10 μM) for the indicated periods of time. The data represent the permeability changes obtained in a representative experiment and are expressed as the mean fold increase (± SEM) in treated cells relative to untreated cells (n=4 for each conditions). p value was determined by using unpaired Student *t* test. **B**) Exponentially growing HUVECs were left untreated (a) or were treated with H_2_O_2_ (250 μM; b) or histamine (10 μM; c) for 30 minutes. Cells were then fixed, were permeabilized, and were stained for F-actin using Alexa 488-phalloidin. A representative field is shown for each condition. Pictures were captured using a Nikon Eclipse 600 fluorescence microscope (40X).

We first ascertained whether oxidative stress induced an increase in endothelial cell permeability. HUVEC cultivated as tight monolayers in Boyden chambers were treated or not with 250 μM H_2_O_2_ for 30, 60 and 90 minutes. Thereafter, permeability was determined by measuring the passage of FITC-labelled dextran (molecular mass: 40 kDa) across endothelial cell monolayers. Results showed that H_2_O_2_ as well as histamine, taken as a positive control, increased endothelial permeability in a time-dependent manner (Figure 1A). In both cases, ROS- and histamine-induced increase in endothelial cell permeability was associated with formation of actin stress fibers, which is consistent with the key role played by these structures in regulating endothelial cell integrity (Figure 1B). Interestingly, the cytoskeletal remodelling induced by H_2_O_2_ and histamine was accompanied by an increase in the width of interendothelial spaces, which reflects cellular contraction that favours permeability to Dextran (Figure 1B). However, the 30 min treatment with 250 μM H_2_O_2_ did not influence cell viability as measured by the cleavage of WST-1 tetrazolium salt in formazan by mitochondrial dehydrogenase activity of viable cells (Additional file 1: Figure S1).

**Figure 2:**
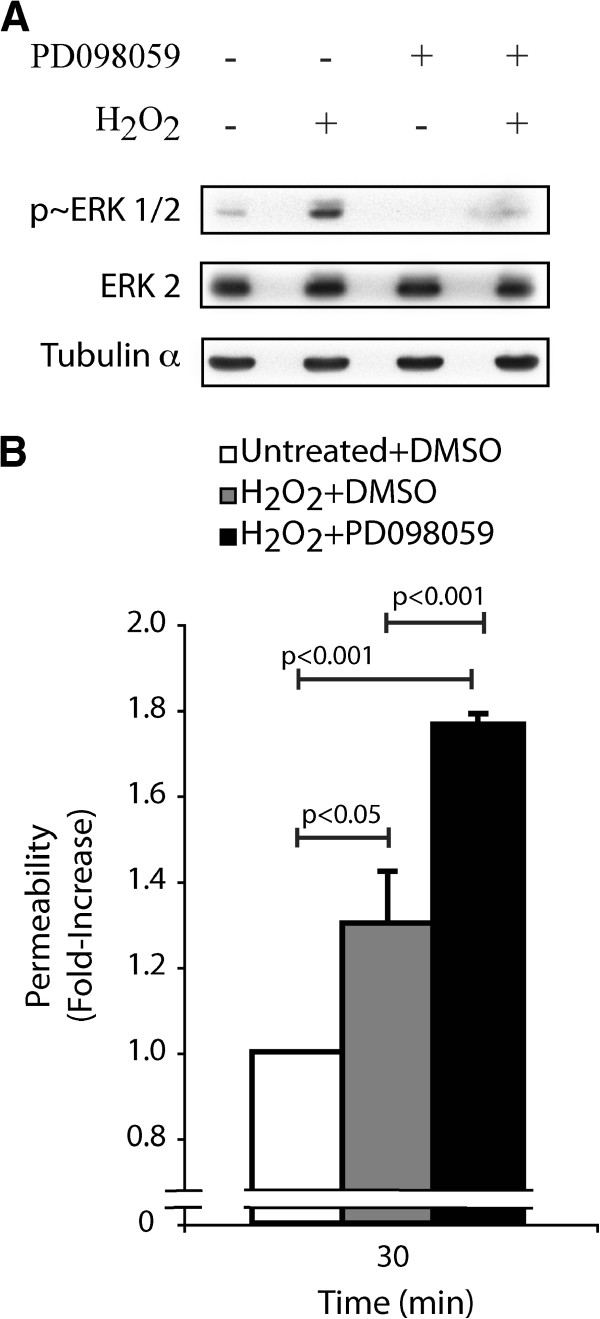
**Inhibition of the ERK MAP kinase in the presence of H**_**2**_**O**_**2**_**is associated with an increase of endothelial permeability.A**) Exponentially growing HUVECs were pretreated with vehicle (0.25% DMSO) or with PD098059 (50 μM) for 60 minutes. Thereafter, HUVECs were treated or not with H_2_O_2_ (250 μM) for 30 minutes. Proteins were extracted, were separated by SDS-PAGE, and were transferred onto nitrocellulose membrane. Inhibition of the ERK pathway was revealed by western blot using phospho-ERK specific antibody (upper panel). Total ERK (ERK2) and tubulin-α expression levels were determined as loading controls (middle and lower panels, respectively). A representative blot is shown **B**) HUVECs were plated at a density of 0.6×10^5^ per well in the upper part of a Boyden chamber and were cultivated for 3 days until the formation of a tight monolayer. Then, the monolayers were pretreated with vehicle (0.25% DMSO) or with PD098059 (50 μM) for 60 minutes, and then they were treated or not with H_2_O_2_ (250 μM) for 30 minutes. Passage of FITC-labelled dextran through the monolayer of HUVECs was measured immediately after treatment. The results represent the permeability changes obtained in a representative experiment and are expressed as the mean fold increases (± SEM) in treated cells relative to untreated cells (n=3). p value was determined by using unpaired Student *t* test.

Next, we evaluated the role of the ERK pathway in maintaining endothelial integrity in response to H_2_O_2_. First, we found that H_2_O_2_ enhanced by 4-fold the activation of ERK after 30 min (Figure 2A). Then, we set up an endothelial permeability assay in which we inhibited the ERK pathway at the level of MEK1/2 using PD098059 [29] (Figure 2A). Results showed that the H_2_O_2_-induced endothelial cell permeability was still further increased by 1.4-fold at 30 min when ERK was inhibited. Indeed, the increased endothelial cell permeability when the ERK pathway was inhibited was augmented by 1.8-fold in comparison to 1.3-fold when the ERK pathway was functional (Figure 2B). Interestingly, PD098059 did not affect by itself endothelial cell permeability for exposure times up to 90 min (Additional file 1: Figure S2).

These findings are strong evidence in support that the ERK pathway is a major regulator of endothelial permeability and that its deregulation in the presence of ROS is associated with disruption of the endothelial barrier functions.

### Tropomyosin-1 regulates endothelial barrier integrity in response to oxidative stress

**Figure 3:**
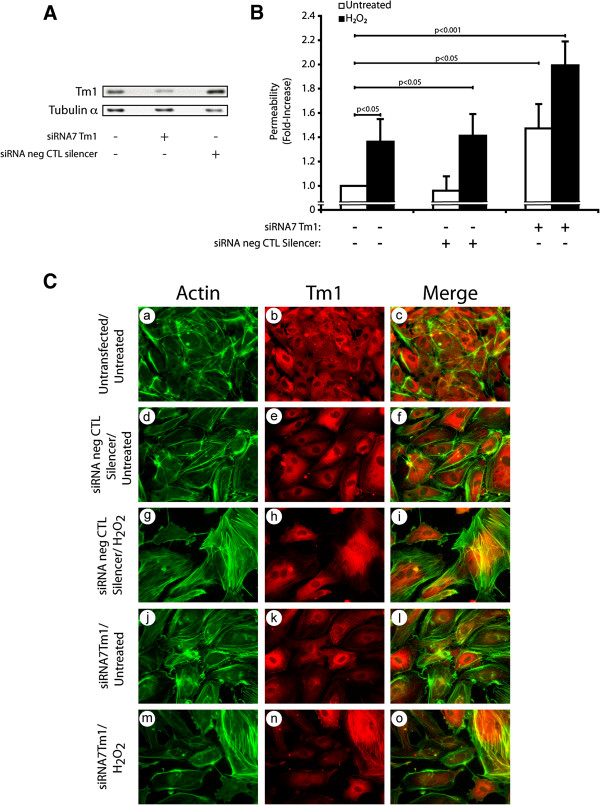
**The knockdown of human Tm1 induces an increase in endothelial permeability in response to H**_**2**_**O**_**2**_**.A**) HUVECs were electroporated or not with Hs_TPM1_7 siRNA (siRNA7Tm1) or non-targeting silencer negative control siRNA. Three days later, proteins were extracted, were separated by SDS-PAGE, and were transferred onto nitrocellulose membrane. Tm1 level was revealed by western blot using anti-tropomyosin mouse monoclonal antibody (upper panel). Tubulin-α level was measured as loading control (lower panel). The siRNA7Tm1-mediated knockdown of Tm1 has been evaluated as 80% following densitometric quantification of the bands. A representative blot is shown. **B**) HUVECs transfected as in **A** using X-tremeGene HP Transfection Reagent were plated at a density of 0.6×10^5^ per well in the upper part of a Boyden chamber and were grown for 3 days until the formation of a tight monolayer. Passage of FITC-labelled dextran through the monolayer of HUVECs was measured after exposure to H_2_O_2_ (250 μM) for 30 minutes. The data represent permeability expressed as the mean fold increases in treated cells (± SEM) relative to untreated cells (n=3 for each conditions). p value was determined by using unpaired Student *t* test **C**) HUVECs were transfected or not (a-c) with siRNAs that specifically target human Tm1 mRNA (j-o) or with non-targeting negative control siRNA (d-i). The next day, HUVECs were plated at a density of 2×10^5^ cells on gelatin-coated microscope cover glasses into 35 mm Petri dish for each condition and were grown for 2 days until the formation of a tight monolayer. Thereafter, cells were treated or not with H_2_O_2_ (250 μM) for 30 minutes (g-i and m-o). Then, cells were fixed, were permeabilized, and were stained for F-actin using Alexa 488-phalloidin (a, d, g, j and m), and for Tm1 using anti-tropomyosin mouse monoclonal antibody (b, e, h, k and n). A merged picture is shown for each condition (c, f, i, l and o). A representative field for each condition was captured using a Nikon Eclipse 600 fluorescence microscope (40X).

We previously reported that ROS-induced phosphorylation of Tm1 at Ser283 by DAP Kinase-1, downstream of ERK, is required to secure the formation of stress fibers and protects endothelial cell against early membrane blebbing [19,21]. Based on these findings, we decided to investigate next the involvement of Tm1 in maintaining the endothelial barrier integrity in response to oxidative stress. To this end, we proceeded to experiments in which we knocked down the expression of endogenous Tm1 by means of siRNAs (Figure 3A and Additional file 1: Figure S3). HUVECs were co-transfected for 72 hours with siRNA7Tm1 or with a non-targeting siRNA (Silencer, Negative Control #1). Monolayers of endothelial cells were treated with 250 μM H_2_O_2_ for 30 minutes and permeability was assessed as above. As shown in Figure 3A and Additional file 1: Figure S3, siRNA7Tm1 almost completely inhibited (by 80%) the expression of Tm1, as determined by densitometric analysis of the western blots performed on cell samples extracted just before the permeability assays. This siRNA-mediated knockdown of Tm1 was associated with an ~2.0-fold increase in endothelial permeability to H_2_O_2_ (Figure 3B). In contrast, the endothelial permeability to FITC-labelled dextran was only ~1.4-fold in H_2_O_2_-treated HUVEC monolayers expressing non-targeting siRNA. Of note, the knockdown of Tm1 by itself was associated with a ~1.4-fold increase in endothelial cell permeability, which is associated with a decreased formation of stress fibers (Figure 3C, Panel j-l). In both cases, this is in line with the fact that Tm1 is an essential component of the endothelial cell integrity. Consistent with the role of Tm1 in stress fiber formation and with the contribution of these latter in maintaining the integrity of the barrier, we found that the knockdown of Tm1 was associated with a loss of stress fibers and with interendothelial spaces when the cells were treated with 250 μM H_2_O_2_ for 30 minutes (Figure 3C: see notably panels j-l vs m-o).

Together, these results constitute the first proof indicating that endogenous human Tm1 is required to maintain the integrity of the endothelial layer in response to ROS.

### Phosphorylation of Tm1 at Ser283 regulates endothelial permeability

**Figure 4:**
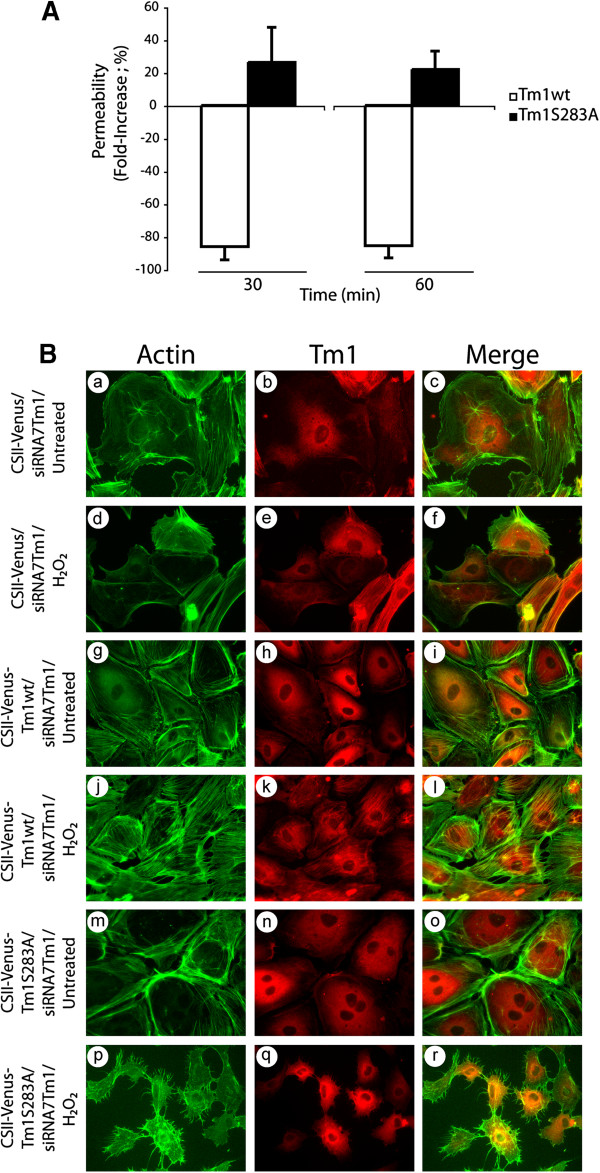
**Phosphorylation of Tm1 at Ser283 regulates endothelial permeability.** Exponentially growing HUVECs were transduced or not with CSII-Venus (empty vector; MOI of 30.2) or CSII-Venus-Tm1wt (Tm1 wild-type vector; MOI of 7.59) or CSII-Venus-Tm1S283A (non-phosphorylatable Tm1 mutant vector; MOI of 6.66) using lentivirus-mediated infection. These Tm1 constructs are insensitive to siRNA7Tm1. After 24 hours, infected HUVECs were transfected with siRNA7Tm1. In **A**) HUVECs were plated at a density of 1x10^5^ cells per well in the upper part of a Boyden chamber and were cultivated for 3 days until the formation of a tight monolayer. Thereafter, NaF (1 mM for 1 hour) was added to the incubation medium (upper chamber) to inhibit phosphatases and to allow the accumulation of phosphorylated tropomyosin-1. Then, H_2_O_2_ (250 μM for 30 or 60 minutes) was added to the upper chamber in the presence of 1 mg/ml FITC-labelled dextran. Passage of FITC-labelled dextran through the monolayer of HUVECs was measured immediately after exposure of HUVECs to H_2_O_2_. The data represent permeability expressed as the mean fold increase (± SEM) relative to untreated cells (n=4 for each condition obtained from a representative experiment out of 4). p value was determined by using unpaired Student *t* test. In **B**) HUVECs were plated at a density of 2×10^5^ cells on gelatin-coated microscope cover glasses into 35 mm Petri dish per condition and were cultivated for 2 days until the formation of a tight monolayer. Thereafter, cells were treated with H_2_O_2_ (250 μM) for 30 minutes (d-f, j-l and p-r) or were left untreated (a-c, g-i and m-o). Then, cells were fixed, were permeabilized, and were stained for F-actin using Alexa 488-phalloidin (a, d, g, j, m and p), and for Tm1 using anti-tropomyosin monoclonal mouse antibody(b, e, h, k, n and q). A merged picture is shown for each condition (c, f, i, l, o, and r). A representative field for each condition was captured using a Nikon Eclipse 600 fluorescence microscope (40X).

The inhibition of the ERK pathway is associated with a decrease in Tm1 phosphorylation at Ser283, which is accompanied by the disappearance of the H_2_O_2_-mediated formation of stress fibers [19,21]. These effects result in breaking the delicate equilibrium between the balanced activation of the ERK and p38 pathways required for the formation of stress fibers and for the normal functioning of endothelial cells in the presence of ROS [18]. In addition, they suggest that phosphorylation of Tm1 at Ser283 downstream of ERK is important to regulate the selective endothelial permeability under oxidative stress. Accordingly, we next verified whether phosphorylated Tm1 at Ser283 plays a role in permeability regulation in response to H_2_O_2_. To this purpose, we used an approach based on knocking down endogenous Tm1 with siRNA in cell expressing different forms of Tm1-insensitive to the knockdown. Briefly, we first introduced, via lentiviral-mediated infection, a wt form or a Ser283Ala mutated form of Tm1 that are insensitive to siRNA7Tm1 (Additional file 1: Figure S4). Twenty-four hours later, the cells were transfected for another 24 hours with siRNA7Tm1 to knock down endogenous Tm1. Then, endothelial cells were cultivated as monolayers in Boyden chambers, and permeability in the presence or not of 250 μM H_2_O_2_ for 30 and 60 min was assayed as above. As shown in Figure 4A, we found a significant 26% (30 min) and 22% (60 min) increase in endothelial permeability in HUVEC monolayers transduced with CSII-Venus-Tm1S283A. In contrast, there were strong 86% (30 min) and 85% (60 min) decreases of endothelial permeability in HUVEC monolayers transduced with CSII-Venus-Tm1wt. Moreover, consistent with the key role played by phosphorylation of Tm1 at Ser283 for the formation of stress fibers and with the role of these latter in permeability, we found that the introduction of wt form of Tm1 is associated with an increase formation of stress fibers in response to H_2_O_2_ (Figure 4B: panels j-l). In contrast, the introduction of the mutated Tm1S283A is associated with absence of stress fibers (Figure 4B: panels m-o), with interendothelial gaps and thereby increased endothelial permeability to H_2_O_2_ (Figure 4B: panels p-r). Interestingly, our results revealed that lentivirus-mediated infection did not increase by itself endothelial permeability and did not affect cell viability (Additional file 1: Figure S5 and Additional file 1: Figure S6) .

Altogether, these results indicate for the first time that phosphorylation of Tm1 at Ser283 is essential to maintain the normal regulation of endothelial permeability in response to oxidative stress.

### Phosphorylation of Tm1 at Ser283 regulates transendothelial migration of HT-29 colon cancer cells

**Figure 5:**
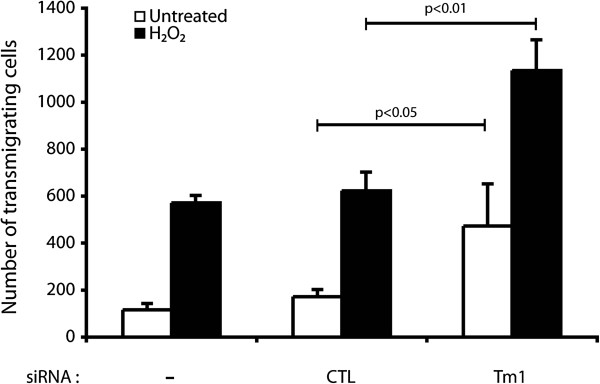
**The knockdown of human Tm1 in endothelial cells is associated with an increase of transendothelial migration of cancer cells.** Exponentially growing HUVECs were transfected or not with siRNA7Tm1 or with non-targeting negative control siRNA. The day after, HUVECs were plated at a density of 0.6×10^5^ per well in the upper part of a Boyden chamber and were cultivated for 3 days until the formation of a tight monolayer. Thereafter, H_2_O_2_ (250 μM) was added or not to the upper chamber for 30 minutes. Then, the medium was removed and replaced by migration buffer. HT-29 cells previously stained with calcein were added on the upper chamber at a density of 1.5×10^5^ cells per well. After 4 hours and a half, HT-29 cells that crossed the membrane of the Boyden chamber were counted in five different fields using a TE300 Nikon fluorescence microscope (20X). In each condition, the number of HT-29 trans-migrating cells was calculated from triplicate samples of a representative experiment (mean±SEM). p value was determined by using unpaired Student *t* test.

Transendothelial migration (TEM) of circulating cancer cells is a key determinant of the metastatic process [16]. Along these lines, TEM and metastasis are both importantly dependent on the integrity of the endothelium and are both affected by ROS [30]. As we reported above, phosphorylation of Tm1 at Ser283 is a major mechanism that confers protection against ROS-induced damage to the endothelial layer. Hence, we next investigated whether phosphorylation of Tm1 at Ser283 regulates TEM under oxidative stress. To ascertain the role of Tm1 in regulating transendothelial permeability under oxidative stress conditions, we first knocked down the expression of endogenous Tm1 via siRNA. Thereafter, cells were cultivated as tight monolayers on a 5.0 μm pore size gelatinized polycarbonate membranes separating the two parts of a 6.5 mm Boyden chambers. The cells were then treated or not with H_2_O_2_ (250 μM) for 30 min. H_2_O_2_-containing media were then removed and were replaced by fresh media containing calcein labeled HT-29 cells in suspension. After 4 hours and a half, the number of HT-29 cells that have crossed the endothelial layer was counted. Consistent with the role of Tm1 in regulating endothelial permeability, we found that transendothelial migration of HT-29 cells was increased by ~2-fold when HT-29 cells were added to a layer of HUVEC in which Tm1 has been knocked down (Figure 5).

**Figure 6:**
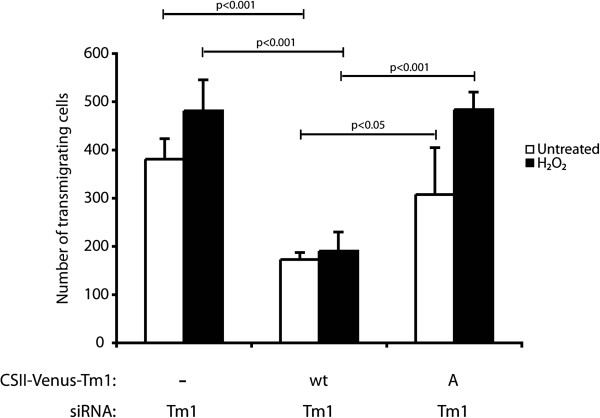
**Phosphorylation of Tm1 at Ser283 is associated with a decrease of cancer cells migration through the endothelial barrier in response to H**_**2**_**O**_**2**_**.** Exponentially growing HUVECs were transduced with CSII-Venus (empty vector; MOI of 30.8) or CSII-Venus-Tm1wt (Tm1 wild-type vector; MOI of 10.4) or CSII-Venus-Tm1S283A (non-phosphorylatable Tm1 mutant vector; MOI of 9.9) using lentivirus-mediated infection. These Tm1 constructs were insensitive to siRNA7Tm1 that was transfected 24 hours later in each condition. The next day, HUVECs were plated at a density of 1.0x10^5^ per well in the upper part of a Boyden chamber and were cultivated for 3 days until the formation of a tight monolayer. Thereafter, H_2_O_2_ (250 μM) was added or not to the upper chamber for 30 minutes. Then, the medium was removed and replaced by migration buffer. HT-29 cells previously stained with calcein were added on the upper chamber at a density of 1.5×10^5^ cells per well. After 4 hours and a half, HT-29 cells that crossed the membrane of the Boyden chamber were counted in five different fields using a TE300 Nikon fluorescence microscope (20X). In each condition, the number of HT-29 trans-migrating cells was calculated from triplicate samples of a representative experiment (mean±SEM). p value was determined by using unpaired Student *t* test.

Given that the Tm1-mediated effect on endothelial integrity depends on its phosphorylation at Ser283, we next verified the role of Tm1 phosphorylation on TEM. The approach used was analogous to that used to investigate the role of Tm1 phosphorylation in regulating endothelial permeability. Briefly, a wild type form or a Ser283Ala mutated form of Tm1 that are insensitive to siRNA7Tm1 were first introduced in the cells, via lentiviral-mediated infection, (Additional file 1: Figure S4). Twenty-four hours later, the cells were transfected for another 24 hours with siRNA7Tm1 to knock down endogenous Tm1. Endothelial cells were then cultivated as monolayers in Boyden chambers and then were treated in the presence or not of H_2_O_2_ (250 μM) for 30 min. Then, media were removed and replaced with fresh media containing HT-29 cells labelled with calcein. After 4 hours and a half, the number of cells that have crossed the layer was counted. The results showed that the re-introduction of the wt form of Tm1 rescued the increased transendothelial permeability in untreated condition as well as in the presence of H_2_O_2_. In contrast, TEM of HT-29 cells was markedly increased in cells expressing Tm1 Ser283Ala. This increase was observed either in endothelial cells that have been treated or not with H_2_O_2_. Yet the effect was more pronounced in this latter case (Figure 6).

Overall, these results are consistent with the hypothesis that increased endothelial permeability is a permissive event for transendothelial migration of cancer cells and that phosphorylation of Tm1 at Ser283 is importantly involved in regulating these events.

## Discussion

It is now accepted that circulating ROS can cause endothelial barrier dysfunction, which may lead to several pathologies, notably atherosclerosis. However, the molecular mechanisms involved are still poorly understood. We previously suggested that the phosphorylation of Tm1 could contribute to maintain endothelial integrity during oxidative stress. In the present study, we now firmly demonstrate the crucial importance of the Tm1 phosphorylation in the regulation of endothelial permeability and transendothelial migration of cancer cells (TEM).

The major finding of our study is that we show that Tm1 expression is important to maintain the endothelial barrier integrity in response to oxidative stress. Two experiments support these new findings. First, permeability assays confirm that the knockdown of Tm1 by small RNA interference in HUVEC monolayers is tightly associated with a heavy increase of endothelial permeability and TEM of cancer cells in response to oxidative stress. Second, in immunofluorescence assays, HUVEC in which Tm1 was decreased with siRNA7Tm1 under oxidative stress conditions display a marked disappearance of stress fibers, a high level of inter endothelial gaps and membrane blebbing.

Another major accomplishment of the present study is to show that Tm1 phosphorylation at Ser283, downstream of ERK is a crucial mechanism that contributes to maintain the selective transendothelial permeability in response to oxidative stress. This finding is supported by two principal observations. First, HUVEC monolayer expressing human non-phosphorylatable Ser283Ala Tm1 insensitive to siRNA7Tm1 and in which human endogenous Tm1 is knocked-down, are characterized by a strong increase of transendothelial permeability under oxidative stress conditions. In contrast, the ectopic expression of human wild-type Tm1 is rather accompanied by an acute decrease of transendothelial permeability in response to oxidative stress. Second, when the expression of human endogenous Tm1 is knocked-down in cells expressing an exogenous nonphosphorylatable Ser283Ala Tm1 insensitive to the knockdown, the endothelium shows important toxic features such as strong shrinkage, high level of inter-endothelial gaps, presence of membrane blebbing, and a total absence of stress fibers. In contrast, the over-expression of human exogenous wild-type Tm1 in similar condition plays a protective role on the endothelial barrier exposed to oxidative stress.

It has been proposed that actin polymerization and remodelling are important regulators of the endothelial permeability barrier [18,31]. Along these lines, our results reveal a strong reorganization of F-actin into transcytoplasmic stress fibers in HUVEC monolayers treated with H_2_O_2_. Almost all cells were elongated with thick actin stress fibers, which traversed the cells in the direction of cell elongation. Moreover, we distinguished an acute presence of inter endothelial gaps suggesting an H_2_O_2_-mediated retraction of the cells. We also noticed the same typical morphological pattern with histamine treatment, a potent inducer of endothelial and vascular permeability [32]. Based on these findings, the H_2_O_2_ and histamine-mediated changes in the organization of actin seem to be a fast and strong response that contributes to increase stress resistance. In this context, the actin depolymerising toxin cytochalasin D decreases endothelial barrier function [33,34]. Hence by its participation to the formation and stabilization of stress fibers, the phosphorylation of Tm1 may regulate the actin-based contraction/retraction of endothelial cells that controls the permeability of the endothelium. Along these lines, other proteins known to modulate actin dynamics and stress fiber formation, such as the small GTPase RhoA, are also known to regulate endothelial cell permeability and TEM [35-37].

The tightness of the vessel walls represents a functional barrier that contributes to limit cancer cell TEM and ultimately metastasis. In that regard, dysfunction of the endothelial integrity is associated with metastasis [16,38,39]. Notably, certain breast, bladder, and kidney cancer cells induce alterations of endothelial mechanical properties to ease their TEM [40]. Given the determinant role played by Tm1 phosphorylation at Ser283 in maintaining the integrity of endothelial cell in response to oxidative stress, one could expect that this mechanism may also protect against metastasis. This is, indeed, supported for the first time by our results. First, in response to H_2_O_2_, TEM of colon cancer HT-29 cells through HUVEC monolayers in which endogenous Tm1 expression is knocked-down by siRNA7Tm1 shows a higher number of transmigrating cells than through control HUVEC monolayer expressing endogenous Tm1. Second, the ectopic expression of human nonphosphorylatable Ser283Ala Tm1 insensitive to siRNA7Tm1 in HUVEC monolayer in which expression of human endogenous Tm1 is knocked-down is associated with an increased level of transendothelial migrating HT-29 cells in response to H_2_O_2_. In contrast, the expression of human exogenous wild-type Tm1 in similar conditions is associated with an acute decreased level of transendothelial migrating HT-29 cells. These results strengthen the point that phosphorylation of Tm1 at Ser283 is crucial to restrict the transendothelial migration of cancer cells under oxidative stress conditions.

In conclusion, we propose that Tm1 is required to maintain the endothelial barrier integrity against molecules as well as against cancer cells both under unstimulated as well as under oxidative stress conditions. We also propose that the phosphorylation of Tm1 on Ser283, downstream of ERK, is a key event for the regulation of endothelial permeability by modulating the contraction/retraction of the endothelial cells via remodelling of the actin cytoskeleton dynamics. These finding strongly support the role for the phosphorylation of Tm1 at Ser283 to prevent endothelial barrier dysfunction associated with oxidative stress.

## Competing interests

The authors declare that they have no competing interests.

## Authors’ contributions

BS have performed the experiments. BS, FH and JH wrote the manuscript. All authors read and approved the final version.

## Authors’ information

Bryan Simoneau, François Houle and Jacques Huot are from « Le Centre de recherche en cancérologie de l’Université Laval and l’Axe Oncologie du Centre de recherche du CHU de Québec, l’Hôtel-Dieu de Québec, 9 rue McMahon, Québec G1R 2 J6 Canada ».

## Supplementary Material

Additional file 1 **Figure S1.** Endothelial cell survival is not decreased by 250 μM H_2_O_2_. HUVECs were cultivated to confluence in a 96-well plates. They were left untreated or were treated for 30 min with 250 μM H_2_O_2_. Medium containing H_2_O_2_ was removed and fresh medium was added. Then, cells were processed for WST-1 assay. Thereafter, absorbance of the samples was determined at 420-480 nm to evaluate the mitochondrial dehydrogenase activity of viable cells as measured by the formation of formazan dye from WST-1. Results are expressed as the mean DO values (+/−SEM) of viable cells from triplicates samples in each condition and are from a representative experiment. p value was determined by using unpaired Student *t* test. **Figure S2**. The MEK1 inhibitor PD098059 does not affect endothelial cell permeability. HUVECs were plated in the upper part of a Boyden chamber at a density of 0.6×10^5^ per well and were cultivated for 3 days until the formation of a tight monolayer. Then, FITC-labelled dextran was added (1 mg/ml) together with DMSO (0.25%) alone or with PD098059 for the indicated period of time. The data represent the permeability changes in the presence of PD098059 over changes by DMSO alone. They were obtained in a representative experiment and are expressed as the mean fold increase (± SEM) in treated cells relative to untreated cells (n=4 for each conditions). p value was determined by using unpaired Student *t* test. **Figure S3**. Hs_TPM1_7 small RNA interference-mediated knockdown of human endogenous Tm1 is effective. A) HUVECs were transfected or not (a-b) with siRNAs that specifically target human Tm1 mRNA (siRNA #7; e-f) or with non-targeting negative control siRNA (c-d) along with plasmid expressing pEGFP-C1. Thereafter, cells were fixed, were permeabilized, and were stained for nuclei using DAPI, for GFP using anti-GFP rabbit antibody, and for Tm1 using an anti-tropomyosin mouse monoclonal antibody. A representative field for each condition was captured using a Nikon Eclipse 600 fluorescence microscope (20X). Arrowheads indicate GFP positive cells. B) Fifteen independent fields from each condition were captured in A, and GFP positive cells were counted. Cells containing a decrease in Tm1 expression were evaluated. The percentage of Tm1 knocked down cells is shown. p value was determined by using unpaired Student *t* test. **Figure S4**. Functionality of lentiviral transgenic expressing vectors (CSII-Venus, CSII-Venus-Tm1wt, and CSII-Venus-Tm1S283A). A) Exponentially growing HUVECs were transduced with CSII-Venus (empty vector; MOI of 30.8) or CSII-Venus-Tm1wt (Tm1 wild-type vector; MOI of 10.4) or CSII-Venus-Tm1S283A (non-phosphorylatable Tm1 mutant vector; MOI of 9.9) using lentivirus-mediated infection. HUVECs were cultivated for 4 days until the formation of a tight monolayer for each condition. Endothelial layers were visualized at 20x magnification by phase contrast (a, d, and g) and by fluorescence microscopy (b, e, and h). A representative field for each condition was captured and a merged picture is shown for each condition (c, f, and i). B) Four independent fields were captured from each lentivirus-mediated infection condition and VENUS positive cells were counted. The percentage of lentiviral-mediated infection from HUVECs monolayer is shown. C) Exponentially growing HUVECs were transduced with CSII-Venus (empty vector; MOI of 30.2) or CSII-Venus-Tm1wt (Tm1 wild-type vector; MOI of 7.59) or CSII-Venus-Tm1S283A (non-phosphorylatable Tm1 mutant vector; MOI of 6.66) using lentivirus-mediated infection. After 24 hours, infected HUVECs were transfected or not with Tm1 siRNA #7. Four days later, proteins were extracted from cells, were separated by SDS-PAGE, and were transferred onto nitrocellulose membrane. Tm1 level was revealed by western blotting using anti-tropomyosin mouse monoclonal antibody (lower panel). Tubulin-α level was measured as loading control (upper panel). D) Relative Tm1 protein levels from C were quantified (Tm1/Tubulin) by densitometry analysis using Image J software and is shown. The experiments are performed at least in triplicates. **Figure S5**. Lentivirus-mediated infection does not increase by itself endothelial permeability. Exponentially growing HUVECs were transduced or not with CSII-Venus (empty vector; MOI of 30.2) using lentivirus-mediated infection. After 24 hours, infected or non-infected HUVECs were transfected or not (controls) with Tm1 siRNA #7. The next day, HUVECs were plated at a density of 1×10^5^ cells per well in the upper part of Boyden chambers and were cultivated for 3 days until the formation of a tight monolayer. Thereafter, NaF (1 mM for 1 hour) was added to the incubation medium (upper chamber) to inhibit phosphatases and to allow the accumulation of phosphorylated tropomyosin-1. Then, H_2_O_2_ (250 μM) was added for 30, 60, and 90 minutes to the upper chamber in the presence of 1 mg/ml FITC-labelled dextran. Passage of FITC-labelled dextran through the monolayer of HUVECs was measured immediately after exposure of HUVECs to H_2_O_2_. The data represent permeability expressed as the mean fold increases (± SEM) relative to untreated cells (n=4). p value was determined by using unpaired Student *t* test. **Figure S6**. Lentivirus-mediated infection does affect endothelial cell survival. Exponentially growing HUVECs were transduced or not with CSII-Venus vectors (empty vector; MOI of 30.8; CSII-Venus-Tm1 wild type vector, MOI of 10.4; CSII-Venus-Tm1S283A mutant vector, MOI of 9.9) using lentivirus-mediated infection. After 24 hours, infected or non-infected HUVECs were cultivated to confluence in a 96-well plates. They were left untreated or were treated for 30 min with 250 μM H_2_O_2_. Medium containing H_2_O_2_ was removed and fresh medium was added. Then, cells were processed for WST-1 assay. Thereafter, absorbance of the samples was determined at 420-480 nm to evaluate the mitochondrial dehydrogenase activity of viable cells as measured by the formation of formazan dye from WST-1. Results are expressed as the mean DO values (+/−SEM) of viable cells from triplicate samples in each condition and are from a representative experiment. p value was determined by using unpaired Student *t* test. Click here for file
