# Supplementary material for: Regulation of endothelial permeability and transendothelial migration of cancer cells by tropomyosin-1 phosphorylation
Source: Vasc Cell. 2012 Nov 17;4:18. doi: 10.1186/2045-824X-4-18 (PMC3552968; doi:10.1186/2045-824X-4-18)
Supplement: Additional file 1 — Figure S1. Endothelial cell survival is not decreased by 250 μM H2O2. HUVECs were cultivated to confluence in a 96-well plates. They were left untreated or were treated for 30 min with 250 μM H2O2. Medium containing H2O2 was removed and fresh medium was added. Then, cells were processed for WST-1 assay. Thereafter, absorbance of the samples was determined at 420-480 nm to evaluate the mitochondrial dehydrogenase activity of viable cells as measured by the formation of formazan dye from WST-1. Results are expressed as the mean DO values (+/−SEM) of viable cells from triplicates samples in each condition and are from a representative experiment. p value was determined by using unpaired Student t test. Figure S2. The MEK1 inhibitor PD098059 does not affect endothelial cell permeability. HUVECs were plated in the upper part of a Boyden chamber at a density of 0.6×10^5^ per well and were cultivated for 3 days until the formation of a tight monolayer. Then, FITC-labelled dextran was added (1 mg/ml) together with DMSO (0.25%) alone or with PD098059 for the indicated period of time. The data represent the permeability changes in the presence of PD098059 over changes by DMSO alone. They were obtained in a representative experiment and are expressed as the mean fold increase (± SEM) in treated cells relative to untreated cells (n=4 for each conditions). p value was determined by using unpaired Student t test. Figure S3. Hs_TPM1_7 small RNA interference-mediated knockdown of human endogenous Tm1 is effective. A) HUVECs were transfected or not (a-b) with siRNAs that specifically target human Tm1 mRNA (siRNA #7; e-f) or with non-targeting negative control siRNA (c-d) along with plasmid expressing pEGFP-C1. Thereafter, cells were fixed, were permeabilized, and were stained for nuclei using DAPI, for GFP using anti-GFP rabbit antibody, and for Tm1 using an anti-tropomyosin mouse monoclonal antibody. A representative field for each condition was captured using a Nikon Eclipse 600 flu [file 2045-824X-4-18-S1.pdf]

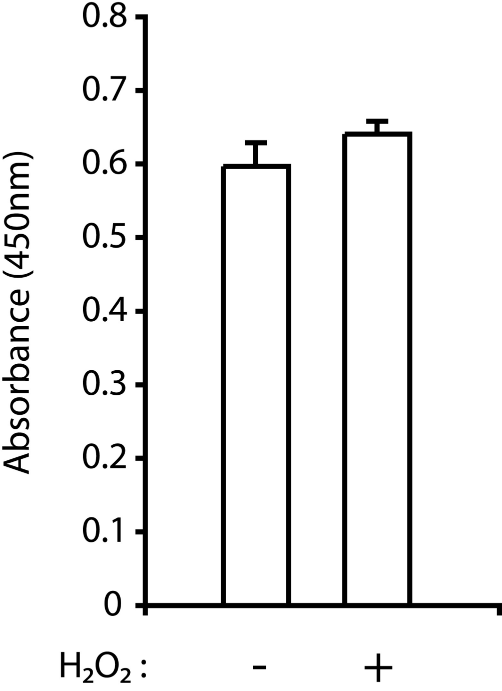

Figure S1.

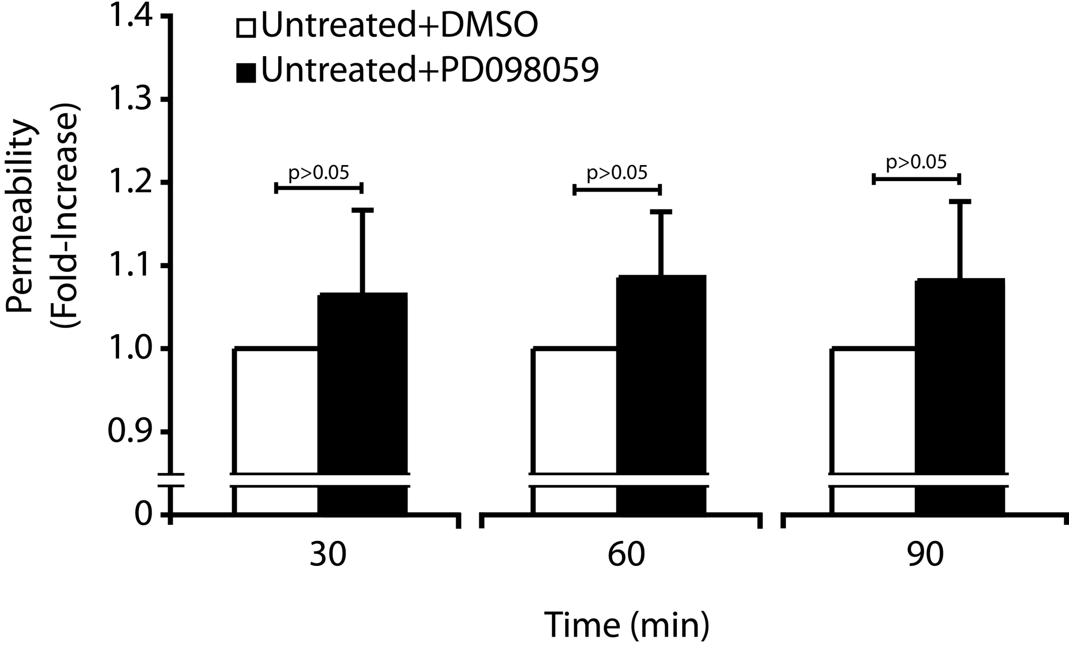

□Untreated+DMSO
■Untreated+PD098059
Permeability (Fold-Increase)
1.4
1.3
1.2
1.1
1.0
0.9
0
p>0.05
p>0.05
p>0.05
30
60
90
Time (min)

Figure S2.

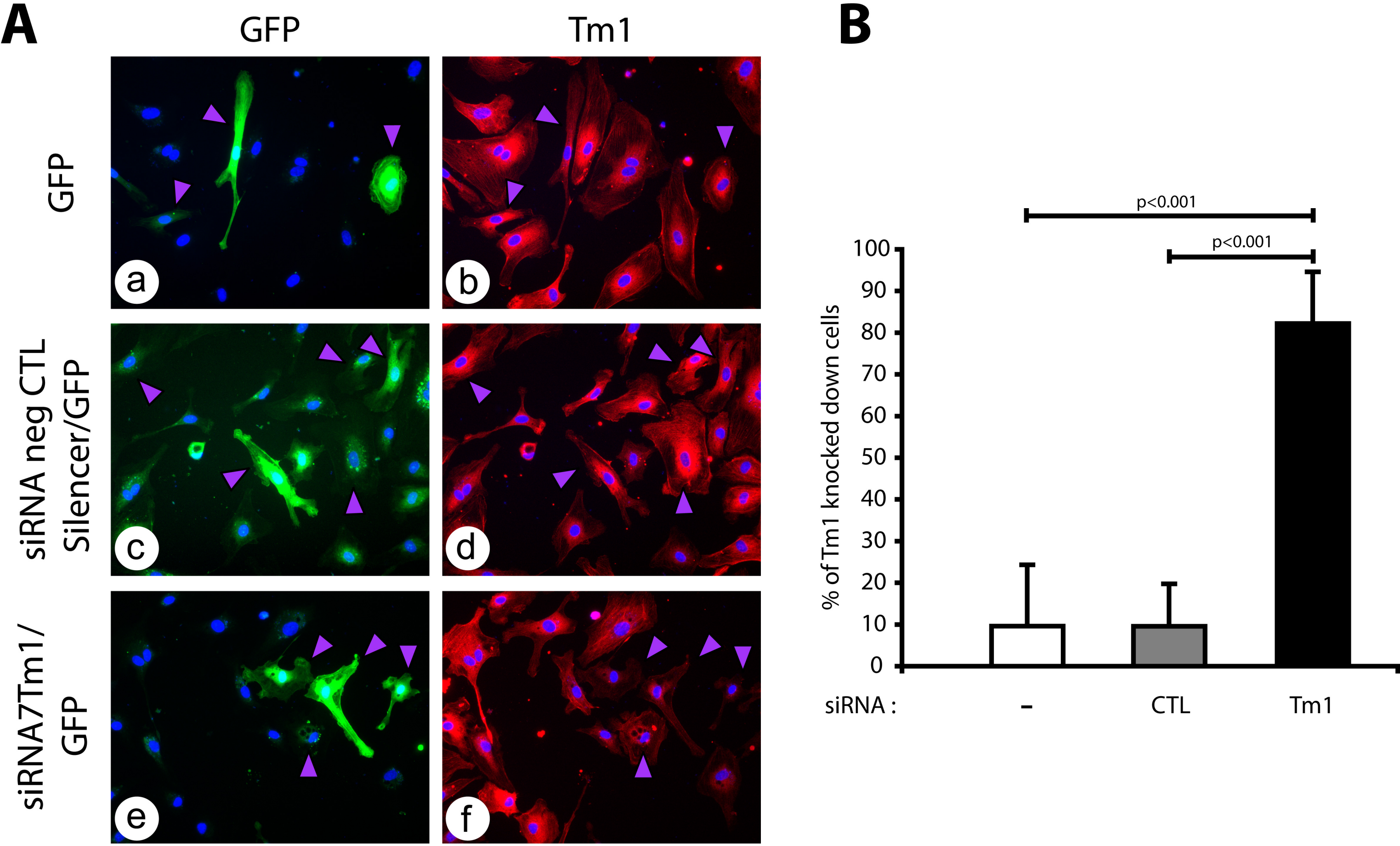
A
GFP
Tm1
GFP
siRNA neg CTL Silencer/GFP
siRNA7Tm1/ GFP
a
b
c
d
e
f
B
p<0.001
p<0.001
% of Tm1 knocked down cells
100
90
80
70
60
50
40
30
20
10
0
siRNA :
-
CTL
Tm1

Figure S3.

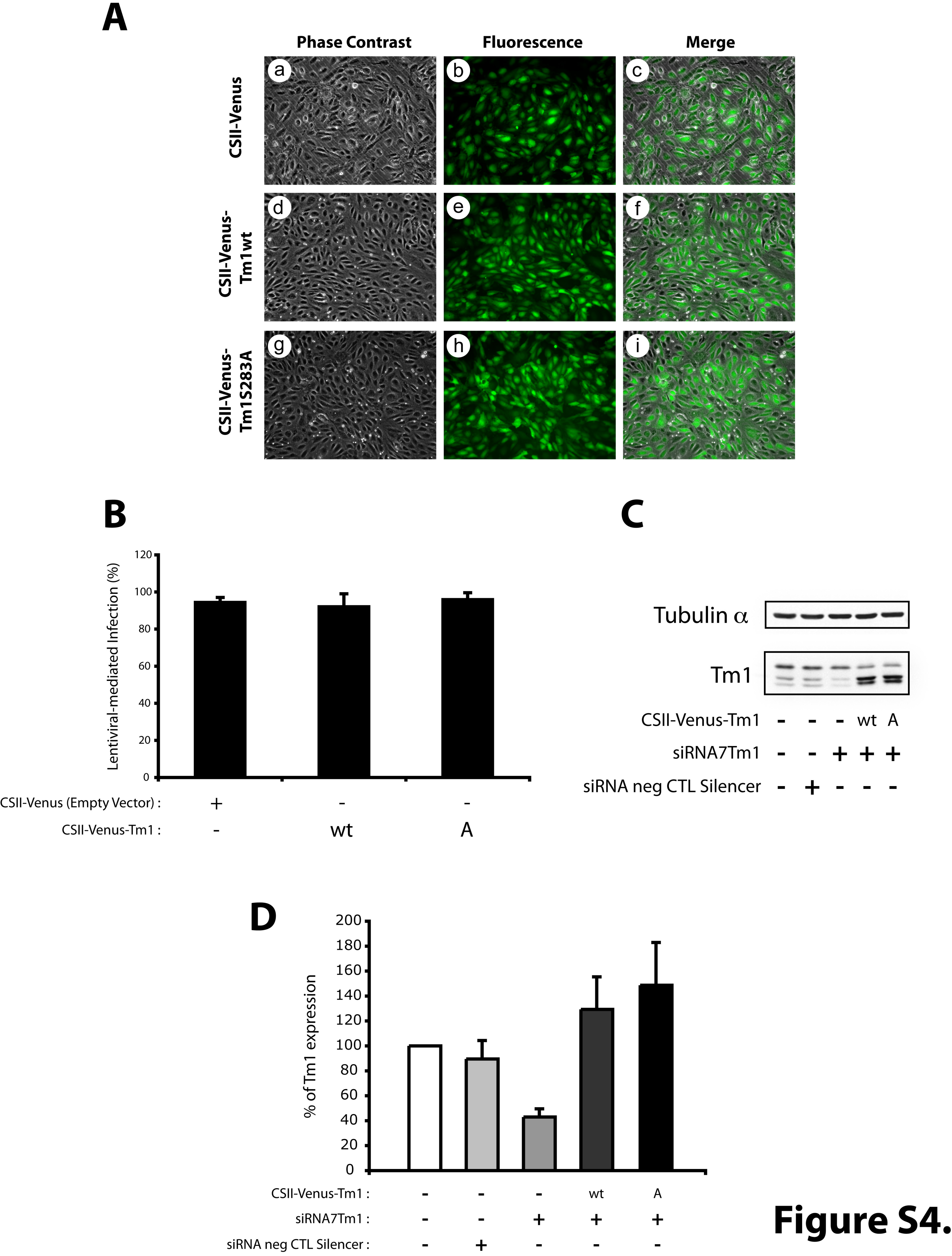
A
Phase Contrast
Fluorescence
Merge
CSII-Venus
CSII-Venus-Tm1wt
CSII-Venus-Tm1S283A
a
b
c
d
e
f
g
h
i
B
Lentiviral-mediated Infection (%)
0
20
40
60
80
100
120
CSII-Venus (Empty Vector) : + - -
CSII-Venus-Tm1 : - wt A
C
Tubulin α
Tm1
CSII-Venus-Tm1 - - - wt A
siRNA7Tm1 - - + + +
siRNA neg CTL Silencer - + - - -
D
% of Tm1 expression
0
20
40
60
80
100
120
140
160
180
200
CSII-Venus-Tm1 : - - - wt A
siRNA7Tm1 : - - + + +
siRNA neg CTL Silencer : - + - - -

Figure S4.

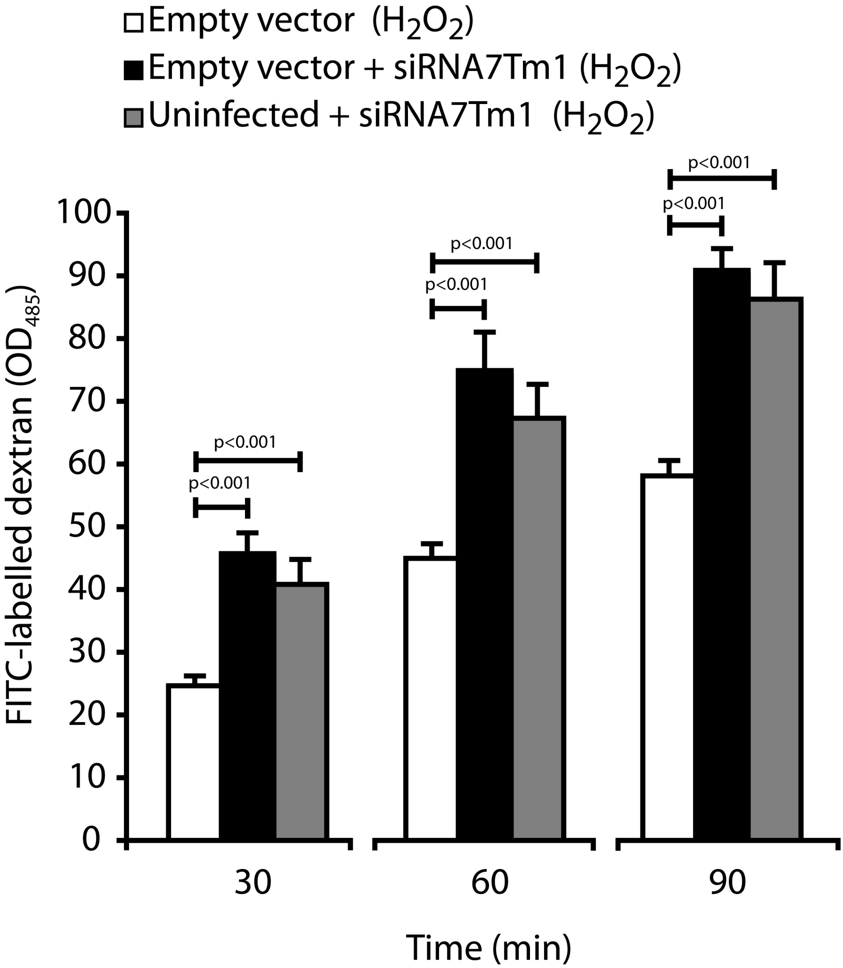
Empty vector (H2O2)
Empty vector + siRNA7Tm1 (H2O2)
Uninfected + siRNA7Tm1 (H2O2)
FITC-labelled dextran (OD485)
100
90
80
70
60
50
40
30
20
10
0
p<0.001
p<0.001
p<0.001
p<0.001
p<0.001
p<0.001
30
60
90
Time (min)

Figure S5.

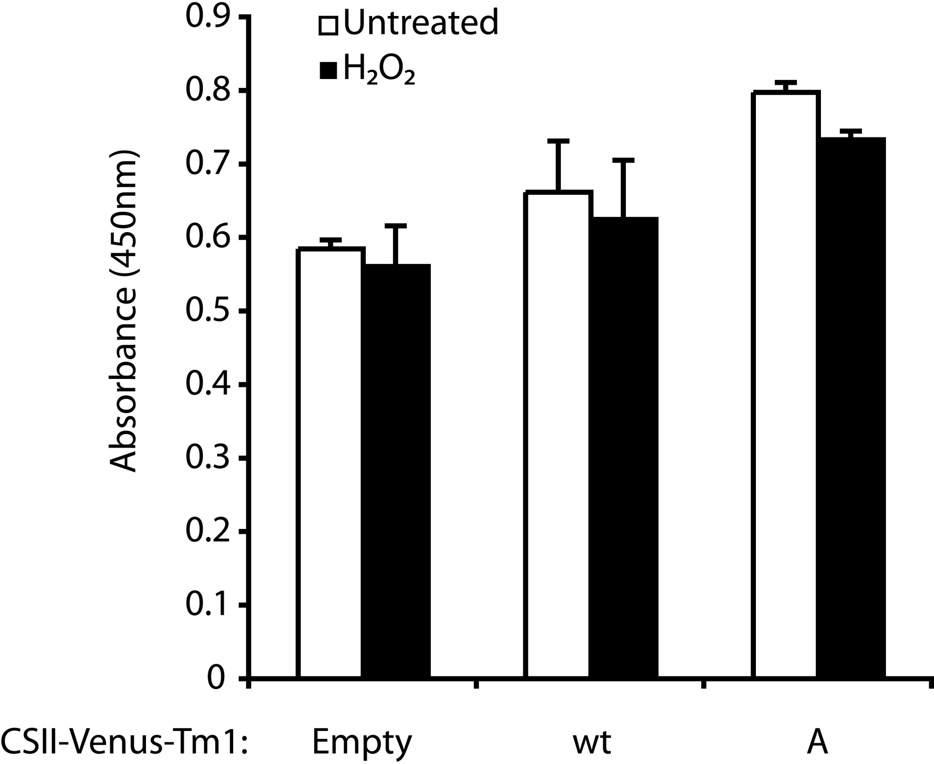

Untreated
H_2O_2
Absorbance (450nm)
0.9
0.8
0.7
0.6
0.5
0.4
0.3
0.2
0.1
0
CSII-Venus-Tm1:
Empty
wt
A

Figure S6.
